# Neural Network Emulation of the Integral Equation Model with Multiple Scattering

**DOI:** 10.3390/s91008109

**Published:** 2009-10-15

**Authors:** Luca Pulvirenti, Francesca Ticconi, Nazzareno Pierdicca

**Affiliations:** Department of Electronic Engineering, Sapienza University of Rome, via Eudossiana 18, 00184 Rome, Italy; E-Mails: ticconi@die.uniroma1.it (F.T.); pierdicca@die.uniroma1.it (N.P.)

**Keywords:** neural networks, surface scattering, radar sensors

## Abstract

The Integral Equation Model with multiple scattering (IEMM) represents a well-established method that provides a theoretical framework for the scattering of electromagnetic waves from rough surfaces. A critical aspect is the long computational time required to run such a complex model. To deal with this problem, a neural network technique is proposed in this work. In particular, we have adopted neural networks to reproduce the backscattering coefficients predicted by IEMM at L- and C-bands, thus making reference to presently operative satellite radar sensors, *i.e.*, that aboard ERS-2, ASAR on board ENVISAT (C-band), and PALSAR aboard ALOS (L-band). The neural network-based model has been designed for radar observations of both flat and tilted surfaces, in order to make it applicable for hilly terrains too. The assessment of the proposed approach has been carried out by comparing neural network-derived backscattering coefficients with IEMM-derived ones. Different databases with respect to those employed to train the networks have been used for this purpose. The outcomes seem to prove the feasibility of relying on a neural network approach to efficiently and reliably approximate an electromagnetic model of surface scattering.

## 1. Introduction

Modeling the electromagnetic wave scattering from random rough surfaces is an important issue for remotely sensing both land (e.g., soil moisture and roughness) and ocean (speed and direction of the wind blowing over the sea surface) geophysical parameters from satellite microwave sensors. As a consequence, a number of theoretical models were developed to deal with this problem. These models necessarily made some simplifying assumptions because of the great complexity of realistic scattering problems [1]. Nevertheless, their implementation generally required performing a large number of calculations, so that the computational efficiency became a critical point, especially if the models were used within inversion algorithms that usually involve repeated runs of the models themselves [2].

If a very rough surface is considered, the phenomenon of multiple scattering should be accounted for [3]. Analytical models such as the Integral Equation Model (IEM) [4,5] have been set up for this purpose. The original formulation [4] had various extensions and updates. Some of these extensions, known as IEMM (Integral Equation Model for Multiple Scattering) [6,7] were just carried out to improve the prediction of the multiple scattering effect. Such an improvement was achieved by removing one of the simplifying assumptions made in [4] (*i.e.*, the use of a simplified expression of the Green's function, see Section 2), so that the complexity of model increased and this increase had repercussions on the problem of its computational efficiency.

An alternative to complex theoretical models is represented by semiempirical techniques. These techniques were widely adopted in the literature, for instance to predict the backscattering coefficient measured by a microwave radar (e.g., a Synthetic Aperture Radar: SAR) aboard satellites or aircrafts (e.g., [8]). They are based on experimental campaigns in which the backscattering radar measurements are coupled with data representing the surface characteristics. The database formed by the data acquired during the campaigns can be analyzed by means of a regression approach to derive a relationship yielding the sensor measurement as a function of the sensor characteristics (frequency, observation angle, polarization) and of some quantities representing the soil conditions, usually expressed in terms of dielectric (e.g., soil moisture) and roughness (standard deviation of heights and correlation length) parameters. The regression analysis permits deriving a simple formula, so that the advantage of semiempirical models in terms of simplicity is evident with respect to physically-based approaches. A critical point is the representativeness of the experimental database, *i.e.*, its ability to encompass a wide set of soil conditions, thus ensuring a large range of applicability of the derived relationship [9].

From the previous discussion, the need to join the simplicity and the efficiency of the semiempirical backscattering models to the precision of physical ones clearly emerges. To succeed in combining these two key features, a neural network approach can be attempted. Since a multilayer feed-forward neural network (NN), having at least one hidden layer, can approximate any nonlinear function relating inputs to outputs [10], it can be profitably adopted to emulate a forward electromagnetic model giving advantages in terms of computational speed and maintaining a fairly good degree of accuracy. The adoption of a NN technique to improve the efficiency of forward models was applied in [11,12] in order to approximate sea surface scattering models.

In this work, a neural network approach to the problem of reproducing the behavior of the IEMM is proposed. We have considered only the backscattering case, because the radar sensors presently operative are monostatic systems, although bistatic experiments have been recently envisaged (e.g., [13,14]). We have made reference to two sensors with different characteristics. The first one is a SAR operating at C-band (5.3 GHz) with an incidence angle *θ_I_* = 23°, such as ERS-2 and also ENVISAT/ASAR in some of its acquisition modes. The second radar configuration, is an L-band (1.25 GHz) instrument with *θ_i_* = 34°, similar to ALOS/PALSAR in fine beam modes. We have firstly built two training sets and two test databases (one for each frequency band) consisting of matched pairs of vectors of input soil parameters (*i.e.*, soil moisture *m_v_*, standard deviation of heights *s* and correlation length *l*) and IEMM outputs (*i.e.*, backscattering coefficients denoted as *σ*^0^). The incidence angles previously mentioned (hereafter denoted also as nominal incidence angles) have been considered in this case. Successively, a second exercise has been carried out in which the incidence angle has been assumed as additional input parameter in order to make the NN-based model applicable for simulating observations of terrains with complex topography. Other four databases (both training and test sets for the two frequencies) have been set up for this purpose. The validation of our method has been carried out by comparing, for the test databases, the IEMM-derived *σ*^0^ with the NN-derived ones.

In Section 2, a summary of the IEMM is provided, while Section 3 introduces the algorithm that has been selected to train the networks, gives some details about the various databases we have built to train and test the behavior of the networks, and describes the design of the NNs architecture. In Section 4, the results are discussed by assessing the simulations of the backscattering coefficients obtained by running the trained NNs against the IEMM outputs. Section 5 draws the main conclusions.

## 2. The Integral Equation Model with Multiple Scattering (IEMM)

The IEMM can be considered as an extension of the Integral Equation based surface scattering model (IEM). With respect to the latter, the IEMM removes the assumption on the phase factor exp(*jw*|*z* − *z*′|), which was neglected in the spectral representations of the Green's function and of its gradient in the development of the original IEM formulation. The quantity denoted by *w* is the vertical component of the propagation vector of the generic plane wave in which the electromagnetic field is expanded, *j* denotes imaginary unit and *z* and *z*′ are the random variables representing the heights at different locations, defined by (*x,y*) and (*x*′,*y*′), respectively, on the rough surface. This approximation was basically thought in order to obtain a simple algebraic form for the scattering model. It was made basing on the small impact of this phase factor on the total average scattered power [4]. However, this factor was shown to be a key element in considering the multiple scattering phenomenon, so that it cannot be ignored [6,7]. In addition, the phase factor in the Green's function with the absolute value sign and an associated time-varying phase of exp(*jωt*), where *t* denotes time and *ω* is the pulsation, indicates that there are two separate cases to consider that correspond to an upward propagation from *z*′ to *z* (*z* > *z*′) and to a downward propagation from *z*′ to *z* (*z* < *z*′).

IEMM expresses the total scattered field as the sum of a term derived from the Kirchhoff tangent plane approximation [15] (Kirchhoff approach) and of a complementary term. Let us consider a Cartesian coordinate system defined by the unit vectors (*x̂,ŷ,ẑ*). The two components of the total electromagnetic field are expressed as:

(1) $$E_{qp}^{k}=CE_{0}\int f_{qp}e^{j\left(k_{s}-k_{i}\right)\cdot r}\text{dxdy}$$

(2) $$E_{qp}^{c}=\frac{C}{8\pi^{2}}\int {\tilde{F}}_{qp}e^{jk_{s}\cdot r}\text{dxdy}=\frac{CE_{0}}{8\pi^{2}}\int F_{qp}e^{ju\left(x-x^{\prime }\right)+jv\left(y-y^{\prime }\right)-jw\left|z-z^{\prime }\right|+jk_{s}\cdot r-jk_{i}\cdot r\prime }\text{dxdydudvd}x^{\prime }dy^{\prime }$$

where *E*_0_ is the incident field amplitude, superscripts *k* and *c* indicate Kirchhoff and complementary terms, respectively, and subscripts *p* and *q* denote the incident and receiving polarizations respectively [6]. **r** and **r**′ represent the observation vectors associated to (*x,y,z*) and (*x*′,*y*′,*z*′), respectively, and (*u,v,w*) are the variables that correspond to (*x,y,z*) in the spectral domain. Moreover:

(3) $$C=-\frac{jk_{0}}{4\pi R}e^{-jk_{0}R}$$

In (3), *k*_0_ is the electromagnetic wavenumber and *R* is the range from the centre of the illuminated area to the point of observation. The scattering (**k***_s_*) and incident (**k***_i_*) propagation vectors are:

(4) $$k_{s}=k_{0}{\hat{k}}_{s}=k_{0}(\hat{x}\sin\theta_{s}\cos\phi_{s}+\hat{y}\sin\theta_{s}\sin\phi_{s}+\hat{z}\cos\theta_{s})$$

(5) $$k_{i}=k_{0}{\hat{k}}_{i}=k_{0}(\hat{x}\sin\theta_{i}\cos\phi_{i}+\hat{y}\sin\theta_{i}\sin\phi_{i}+\hat{z}\cos\theta_{i})$$

where *θ* and *φ* are the zenith and azimuth angles, subscripts *i* and *s* indicate incidence and scattering directions, respectively, *k̂_s_* and *k̂_i_* are the unit vectors in the direction of scattering and incidence, respectively. The Kirchhoff and complementary field coefficients, *f_qp_* and *F̃_qp_* respectively, are dimensionless, complicated expressions that depend on spatial variables:

(6) $$f_{qp}=[\hat{q}\times k_{s}\cdot {\left(\hat{n}\times E_{p}\right)}^{k}+\eta \hat{q}\cdot {\left(\hat{n}\times H_{p}\right)}^{k}]D_{1}/E_{i}$$

(7) $${\tilde{F}}_{qp}=8\pi^{2}[\hat{q}\times k_{s}\cdot {\left(\hat{n}\times E_{p}\right)}^{c}+\eta \hat{q}\cdot {\left(\hat{n}\times H_{p}\right)}^{c}]D_{1}$$

(8) $$D_{1}=\sqrt{1+{\left(dz/dx\right)}^{2}+{\left(dz/dy\right)}^{2}}$$

where (*n̂* × **E***_p_*)*^k^* and (*n̂* × **H***_p_*)*^k^* are the tangential components of the Kirchhoff field at *p* polarization, while (*n̂* × **E***_p_*)*^c^* and (*n̂* × **H***_p_*)*^k^* are the tangential components of the complementary field (*p* polarization). *n̂* is the unit vector normal to the surface, *q̂* is the observed polarization versor (either horizontal, or vertical) and *η* is the intrinsic impedance in air. The incident electric field is written as **E***_i_* = *p̂E*_0_*e*^−jk0^**^ki·r^**, where *p̂* is the incident polarization versor. From (1) and (2), the backscattering coefficient can be determined, being defined as:

(9) $$\sigma_{pq}^{0}=\frac{4\pi R^{2}\langle {\left|E_{qp}\right|}^{2}\rangle }{A{\left|E_{i}\right|}^{2}}$$

where *E_qp_* is the sum of (1) and (2), 〈·〉 denotes ensemble average and *A* is the illuminated area.

Several approximations were accomplished to make *f_qp_* and *F̃_qp_* independent of spatial variables. The expression of *f_qp_* is in general very complicated and depends on local surface slopes and local Fresnel reflection coefficients [4,5]. In IEM, a good approximate expression was derived for *f_qp_* and was also used in the IEMM. The complementary field coefficients *F_qp_* that appear in the right term of Equation (2) were obtained from the *F̃_qp_* after the Green's function and its gradient were replaced by the spectral representation that takes into account the inclusion of the phase factor, exp(*jw*|*z*−*z*′|), and after the phase factor of the Green's function and *u, v, x*′, *y*′ integrations were factored out. The introduction of this phase factor allowed splitting the coefficients into two parts, namely the upward complementary field coefficients *F_qp,up_* and the downward complementary field coefficients *F_qp,down_*. The resultant expressions are reported in [6].

In [6], the backscattering coefficient from a perfectly conducting Gaussian correlated surface was simulated through the IEMM model both considering and not considering the multiple terms. The results showed that the multiple scattering effect is very different for the co-polarized horizontal and vertical polarizations. Indeed, it is highly sensitive to the wave polarization states. The frequency backscattering coefficients behavior was also analyzed. At lower frequencies and smaller incident angles, the multiple scattering has little contributions; results of the IEMM are closely together and agree with the single scattering. As frequency increases, multiple scattering increases its contributions to backscattering coefficient.

We can conclude this brief summary by pointing out that the IEMM model leads to a more accurate calculation of the multiple scattering contribution with respect to IEM [6]. The latter had several improvements in the last fifteen years to better evaluate the scattering from natural surfaces for the various measurement configurations and types of roughness. The last version, known as Advanced Integral Equation Model [16], introduced further modifications on the expression of the scattering complementary field. However, only the single scattering terms were derived again, whereas the multiple-scattering terms were not changed with respect to the formulation developed in the IEMM version.

## 3. The Neural Network Emulators

An artificial NN is a nonlinear parameterized mapping from an input vector **x** to an output vector **y** = NN(**x;w**,*M*), where **w** is the weight vector (including the biases as well) and *M* is the architectural model of the network. The multilayer perceptron (MLP) architecture considered here is a mapping model that is composed of several layers of parallel processors (known as neurons). It was demonstrated that one-hidden layer MLP network can approximate any continuous function [17], while a two-hidden-layer MLP can represent any function to any degree of nonlinearity, taking also into account discontinuities [18].

In the architecture of a NN, all nodes are interconnected to each other, and this interconnection is characterized by weights and biases. The hidden and output nodes are characterized by an activation function, which is generally assumed to be differentiable and nonlinear. Here, we have chosen the tan-sigmoid function, which is characterized by the node gain and the node bias.

### 3.1. The training algorithm

The network is trained by a supervised learning using a training database *D* = {**x**(*i*),**t**(*i*)}, consisting of available inputs **x**(*i*) and desired targets **t**(*i*), being *i* = 1:*N_r_* and *N_r_* the number of the records of the training set. During training the weights and biases are iteratively adjusted in order to minimize the network performance or objective function (error correction learning). The latter is generally assumed equal to the sum of squared errors *E_D_*:

(10) $$E_{D}(w)=\sum_{i=1}^{N_{r}}{\left[t(i)-y(i)\right]}^{T}[t(i)-y(i)]$$

where ***y***(*i*) is the neural network response to the *i*-th input pattern and the superscript *T* indicates matrix transpose.

The minimization with respect to **w** is based on repeated evaluation of the gradient of the performance function using the back-propagation algorithm, which involves performing computations backwards through the network [19]. Although the back-propagation learning rule is often implemented by using a steepest-gradient descent algorithm, we have chosen for our design the Levenberg-Marquardt (LM) algorithm, which turned out to be highly efficient, as proved in [20].

It is worth considering that, when attempting to emulate rough surface scattering models, the NN training set can hardly encompass all the possible model inputs, because of the high variability of surface characteristics (e.g., roughness and soil moisture in our case). The capability of a neural network to properly respond to unexpected inputs is called generalization. The procedure to improve generalization, called regularization, usually adds an additional term to the error objective function. Such a modified function, denoted as *E_R_*, is expressed by:

(11) $$E_{R}(w)=\alpha_{D}E_{D}(w)+\alpha_{W}E_{W}(w)$$

where *E_W_* is the sum of squares of the network weights **w** (*i.e.*, Σ*_i_***w**^T^**w**), while α*_D_* and α*_W_* are the regularization parameters. If α*_D_* ≫ α*_W_* the training algorithm will minimize the NN error term *E_D_*, while if α*_D_* ≪ α*_W_* the algorithm will emphasize weight size reduction, at the expense of larger network errors, producing a smoother, but more robust, network response.

A critical issue of the regularization procedure is the evaluation the optimal values for the regularization parameters α*_D_* and α*_W_*. To tackle this problem in an automatic way, it is possible to rely on the Bayesian theory, as done in [21], in which the weights and biases of the network were supposed to be random variables with specified distributions, while the regularization parameters were related to the unknown variances associated with the distributions. These parameters can be estimated by using statistical techniques. The estimation procedure is fairly complex and its description is beyond the scopes of this work. More details on the use of Bayesian regularization, in combination with Levenberg-Marquardt training, can be found in [22]. Such a combined use has been accomplished to train the NNs we have developed.

### 3.2. The training sets

For each of the two frequency bands considered here, two cases have been considered, so that four NNs have been designed. All the training and test databases have been built by means of the IEMM, whose runs have been performed by adopting an exponential autocorrelation function (ACF). In the first case, for which the incidence angle has been maintained constant (C-band: 23°; L-band: 34°), a training set consisting of *N_r_* = 500 input/output pairs has been produced for each frequency. For this generation, the following input parameters have been used: soil moisture content *m_v_*, standard deviation of heights *s* and correlation length *l*. These inputs have been randomly generated, by assuming a uniform distribution [9] within the following intervals: 0.05 ≤ *m_v_* ≤ 0.4; 6 cm ≤ *l* ≤20 cm; 0.1 ≤ *k*_0_*s* ≤ 3. Note that the limits of those intervals have been chosen to fulfill the validity of the IEMM and that the choice of *k*_0_*s* ≤ 3 justifies the use of the exponential ACF that is generally recognized as the appropriate one if *s* is not very large (in comparison with the wavelength *λ*, being *k*_0_ = 2π/*λ*) [23-25]. As for the outputs, the co-polarized backscattering coefficients at both vertical $\left(\sigma_{vv}^{0}\right)$ and horizontal $\left(\sigma_{hh}^{0}\right)$ polarizations have been considered.

In the second case, the incidence angle has been assumed as an additional parameter to allow the NN-based model to be suitable for terrains with complex topography too. While the soil parameters have been randomly generated with the same limits as those listed above (so that an exponential ACF has been chosen again to run the IEMM), for *θ_i_* we have supposed a displacement in the order of 15° from the nominal value, so that uniform distributions have been considered within the following intervals: 10°–40° (C-band) and 20°–50° (L-band). Such an hypothesis has been formulated in agreement with the results we found in [26] for the standard deviation of the local incidence angle on tilted surfaces. For each frequency, a training set of *N_r_* = 2,000 input/output pairs has been built.

For the soil permittivity, a model proposed in the literature that relates it to soil moisture, temperature and composition has been selected [27]. For the latter quantities, fairly standard values have been considered (soil temperature: 23°; percentages of sand and clay: 48.5% and 12.5%, respectively). Note that a value of 23° for the soil temperature has been chosen in agreement with the measurements of dielectric constant reported in [28], in which it is also claimed that both the real and the imaginary parts of the soil dielectric constant are weakly dependent on temperature as well as on the soil type.

The same procedure followed to produce the training databases has been employed to build the test sets. 500 input/output pairs have been generated (both at C- and L-bands and independently of those produced for the training database) considering the nominal *θ_i_*, while 4,000 records have been generated to assess the behavior of NNs designed for the case in which *θ_i_* is an additional input parameter.

### 3.3. The architectures

The architectures of the NNs designed to reproduce the *σ*^0^ predicted by the IEMM model at C- and L-bands consist of three (corresponding to *m_v_, s* and *l*) or four (*m_v_, s, l* and *θ_i_*) input neurons (*N_i_*) and two output neurons (*N_o_*), corresponding to the co-polarized backscattering coefficients at vertical and horizontal polarizations. As for the hidden layer, that in this case basically accounts for the interaction between the electromagnetic radiation irradiated by the radar antenna and the terrain, we have firstly considered one layer of *N_h_* neurons. To determine *N_h_*, we have monitored both the number of epochs ensuring the convergence of the learning algorithm and the value of the objective function at the final epoch. L-band, for which the emulators yield worse performances with respect to the C-band (see Section 4), has been considered for this purpose. Note that the convergence of the algorithm has been assumed corresponding to the stabilization of the values of the objective function. Table 1 reports the results we have obtained considering the nominal incidence angle.

From Table 1, it can be observed that *E_R_* rapidly decreases with the increase of *N_h_* from 5 to 15, while it tends to become more stable for *N_h_* ≥ 15. Moreover, if *N_h_* increases from 15 to 25, *N_E_* almost doubles and we have verified on the test set that such an increase of *N_h_* does not imply a substantial improvement of the capability of the NN to approximate the behavior of the IEMM.

Considering that a two-hidden-layer MLP network can approximate any function to any degree of nonlinearity (see Section 3) and taking into account the high degree of nonlinearity of a very complex scattering model such as the IEMM, we have also designed an architecture consisting of two hidden layers of *N_h_*_1_ and *N_h_*_2_ neurons, respectively. The results are reported on Table 2, which suggests that the addition of a second hidden layer leads to a substantial improvement of the NN performances in terms of *E_R_*, although *N_E_* considerably increases. The best compromise between the complexity of the architecture and the need to achieve small values of *E_R_* seems to be *N_h_*_1_ = 15 and *N_h_*_2_ = 10.

**Figure 1.**
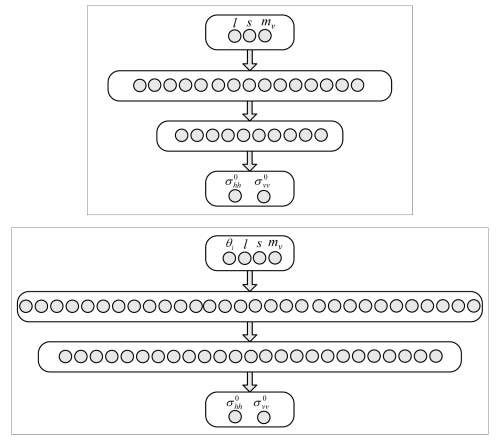
The Neural Network architectures. Upper panel: NN topology for the nominal incidence angle (*N_i_* = 3, *N_h_*_1_ = 15, *N_h_*_2_ = 10, *N_o_* = 2); lower panel: NN topology for incidence angle assumed as an additional input parameter (*N_i_* = 4, *N_h_*_1_ = 30, *N_h_*_2_ = 25, *N_o_* = 2).

The same exercise has been accomplished for the case in which *θ_i_* is included in the inputs of the NN-based model (*i.e., N_i_* = 4). As expected, with one hidden layer the values of *E_R_* have turned out to be fairly large (e.g., 15.8 with *N_h_* = 25). Even with two hidden layers, we have succeeded in achieving values of *E_R_* in the order of those reported on Table 2 only by considerably increasing both *N_h_*_1_ and *N_h_*_2_. By looking at Table 3, that is the same as Table 2, but for *N_i_* = 4, it can be noted that *E_R_* < 0.1 has been obtained for *N_h_*_1_ = 30, *N_h_*_2_ = 25. An architecture defined by: *N_i_* = 3, *N_h_*_1_ = 15, *N_h_*_2_ = 10 and *N_o_* = 2 has been selected for the two networks (L- and C-bands) designed for the nominal *θ_i_*. For variable *θ_i_*, we have set up two networks characterized by *N_i_* = 4, *N_h_*_1_ = 30, *N_h_*_2_ = 25 and *N_o_* = 2. Both kinds of architecture are shown in Figure 1.

Since the activation functions of the hidden layers are tan-sigmoid, and those of the output neurons are linear, these architectures imply that the NN outputs can be expressed by the following relationships:

(12) $$\sigma_{k}^{0}=\left\{ \begin{array}{l} \sum_{h=1}^{10}w_{kh}\left\{\text{tansig}\left[\sum_{j=1}^{15}w_{hj}\left[\mathrm{tansig}\left(\sum_{i=1}^{3}w_{ji}x_{i}+b_{j}\right)\right]+b_{h}\right]\right\}+b_{k}\;\text{for fixed}\;\theta_{i}\\ \sum_{h=1}^{25}w_{kh}\left\{\text{tansig}\left[\sum_{j=1}^{30}w_{hj}\left[\mathrm{tansig}\left(\sum_{i=1}^{4}w_{ji}x_{i}+b_{j}\right)\right]+b_{h}\right]\right\}+b_{k}\;\text{for variable}\;\theta_{i} \end{array}\right.;k=1:2$$

where *w_ji_* and *b_j_* represent weights and biases of the *j*-th neuron of the first hidden layer, *w_hj_* and *b_h_* denote weights and biases of the *h-th* neuron of second hidden layer, and *w_kh_* and *b_k_* indicate weights and biases of the *k-th* neuron of the output layer (*k* = 1 corresponds to *vv; k* = 2 corresponds to *hh*) and the generic input variable is indicated by *x_i_*. Equations (12) can be easily implemented by using the values of weights and biases resulting at the end of the training phase (not reported for the sake of conciseness, but available by contacting the authors). Note that the NNs have been trained after having carried out a normalization aiming at obtaining inputs and targets falling approximately in the range [−1,1].

Before ending this section, it is worth underlining that the generation of the backscattering coefficients by means of the IEMM has taken about 24 hours per 1,000 records (by employing a personal computer with a 3.2 GHz Pentium 4 processor and 2 GB of RAM). The two networks whose architecture is shown in the upper panel of Figure 1 have been trained (with the same computer) in 146 sec (L-band) and in 128 sec (C-band), while the training of the other two NNs (lower panel of Figure 1) has required much more time (L-band: 140 min; C-band: 85 min). Finally, the generation, by means of the trained networks, of the backscattering coefficients used to test the performance of the emulator (see Section 4) has turned out to be almost immediate.

## 4. Results

To assess the proposed approach, we have compared the backscattering coefficients produced by the IEMM model and belonging to the test sets with those generated by the NNs for the same inputs. Note that, as pointed out at the end of Section 3, the trained NNs generate outputs in the interval [−1,1], so that an inverse normalization has been accomplished to restore the nominal range for the backscattering coefficients.

**Figure 2.**
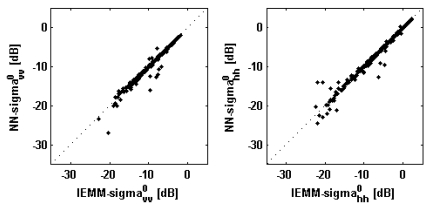

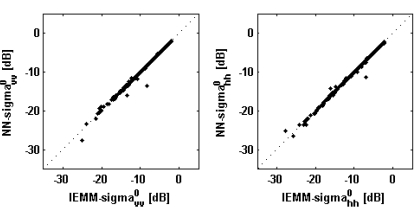
Comparison between IEMM- and NN-derived *σ*^0^ (test sets of 500 records). Left panels: vertical polarization; right panels: horizontal polarization. Upper panels: L-band; lower panels: C-band. Dotted lines represent perfect agreement.

Figure 2 shows the comparison between IEMM- and NN-derived backscattering coefficients for a fixed *θ_i_* equal to the nominal incidence angle. The backscattering coefficients are represented in dB to be consistent with most of the literature works on surface scattering measurements (e.g., [6-8]). The agreement is good at both vertical and horizontal polarizations, especially at C-band, thus assessing the reliability of the proposed approach.

The performances can be also evaluated by looking at Table 4 that reports the results of the comparison in terms of correlation coefficient *ρ*, bias error *b* (*i.e.*, mean error, defining the error as the difference between the *σ*^0^ predicted by NNs and those estimated by IEMM) and root mean square error *rmse. ρ* is close or equal to the unit, while *b* is almost null for both the frequency bands. At C-band the *rmse* is in the order of 0.3 dB, while at L-band it is slightly larger (∼0.7 dB).

**Figure 3.**
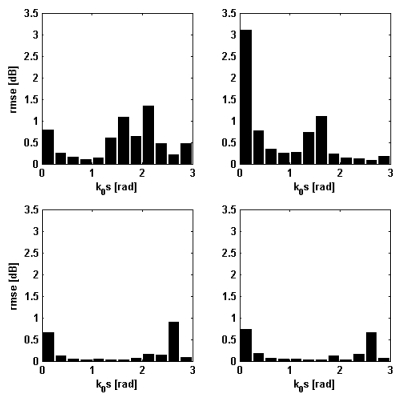
Trend of the *rmse* (error bars) versus *k*_0_*s*. Left panels: vertical polarization; right panels: horizontal polarization. Upper panels: L-band; lower panels: C-band.

Other details on the behavior of the NN-emulators designed for the nominal incidence angle can be inferred by looking at Figure 3 that has been produced by dividing the range of *k*_0_*s* belonging to the test sets (500 records) in intervals of 0.25 rad and by computing, for each interval the *rmse*. This parameter never exceeds 1 dB at C-band, while, at L-band, it is less than 1.5 dB, except for horizontal polarization and *k*_0_*s* around 0.125 rad (see the first bar in the upper right panel of Figure 3). However, we underline that such a smooth kind of soil does not occur very frequently in real situations, so that the overall result can be considered satisfactory. At C-band the largest values of *rmse* have been found either for very smooth or very rough soils, while for fairly standard values of *k*_0_*s, rmse* is very small (0.1–0.2 dB).

**Figure 4.**
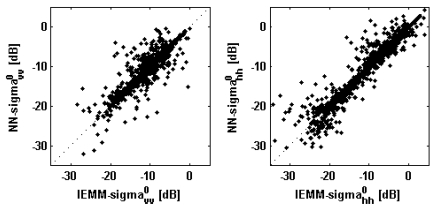

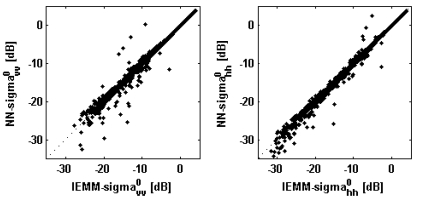
Same as Figure 2, but for *θ_i_* assumed as an additional input parameter (test sets of 4,000 records).

**Figure 5.**
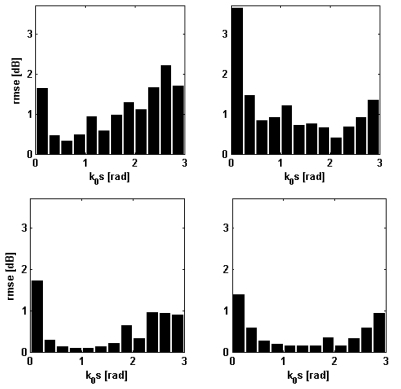
Same as Figure 3, but for *θ_i_* assumed as an additional input parameter.

It can be expected that the NNs designed to include the incidence angle in the input parameters (Figure 1, lower panel) do not provide the same high accuracy as those envisaged for *N_i_* = 3 in reproducing the IEMM behavior. Figure 4, Figure 5 and Table 5 are the same as Figure 2, Figure 3 and Table 4, respectively, but for *θ_i_* assumed as an additional input parameter. We remind that in this case the number of records forming the test databases is 4,000, considerably larger than the dimension of the tests sets considered in the above discussion (500 records). Looking at Figure 4, it can be seen that, with respect to the previous case, the points of the scatterplot fluctuate more around the main diagonal at L-band. The results reported on Table 5 show that *ρ* is still close to the unit, *b* is still almost null, and that *rmse* exceeds 1 dB at L-band. The results can be however evaluated as encouraging considering the complexity of the problem of approximating the IEMM-behavior in this case in which its dependence on the incidence angle should be reproduced besides that on the soil parameters. Such a complexity is testified by the fairly long time required to train the NNs (see Section 3.3).

Comments on Figure 5 are substantially the same as those on Figure 3. The root mean square error is generally less than 1 dB (C-band) and 2 dB (L-band). Again, a large *rmse* has been obtained only for L-band, horizontal polarization, for very small *k*_0_*s*. Note that for the same range of *k*_0_*s* we have found the largest values of *rmse* at C-band too. A similar analysis has been accomplished also for the incidence angle in this case. The range of *θ_i_* has been divided in 5°-step intervals and for each interval the *rmse* has been calculated. The result of this analysis (not shown for the sake of conciseness) has demonstrated that *rmse* is not very sensitive to incidence angle, except for a slight increase occurring for the largest *θ_i_* considered here (*i.e.*, 40° at C-band and 50° at L-band). We can end this discussion by claiming that the NN-based model can be reliably applied to reproduce the behavior of the IEMM (especially at C-band) both for flat and for hilly terrains. Caution must be used in considering the NN predictions at L-band, horizontal polarization, for the case of very smooth soils, because we have found an error in the order of 3 dB.

A final test has been accomplished to assess the reliability of the proposed approach. Considering separately the test sets built for the nominal incidence angle (L-band: 34°; C-band: 23°), we have added to the IEMM-based *σ*^0^ a random Gaussian noise of zero mean and 1 dB of standard deviation, in order to simulate both the model and the instrument errors. Then, we have tried to retrieve the corresponding input *m_v_*, through an iterative technique aiming at minimizing the following cost function:

(13) $$d(m_{v})=\sum_{k=1}^{2}{\left[\sigma_{k\_\text{meas}}^{0}-\sigma_{k\_NN}^{0}(m_{v})\right]}^{2}$$

In (13), *k* = 1 corresponds to *vv* and *q* = 2 corresponds to *hh*. With subscript *meas*, we denote the IEMM *σ*^0^ to which the noise was added thus simulating a radar sensor measurement, while with subscript NN we indicate the outputs of the trained network. The minimization of (13) has been performed by applying a simulated annealing technique (e.g., [29]). Note that since this is only a theoretical exercise aiming at proving the suitability of our methodology for a typical remote sensing problem such as soil moisture estimation, the knowledge of the roughness parameters (*i.e., s* and *l*) has been supposed to generate the NN outputs, while it is well known that in real cases the uncertainty on the roughness parameters affects the quality of soil moisture estimates inducing retrieval errors that may be quite large [30,31].

Table 6 reports the results of the comparison between the estimations achieved by minimizing (13) and the *m_v_* of the test sets. The retrievals obtained by using the NN emulators of the IEMM are fairly accurate (especially at C-band), so that the use of the NN-based forward model, instead of the rigorous IEMM, seems to be promising for an application to the problem of retrieving geophysical parameters from radar data. It is worth noting that, by using the NNs, the minimization has taken approximately 3 minutes for all the 500 samples of the test sets.

## 5. Conclusions

A neural network approach to approximate the behavior of the Integral Eqution Model with multiple scattering (IEMM) has been proposed to deal with the problem of the IEMM computational efficiency. The backscattering coefficients evaluated at C-band, considering an observation angle of 23° and at L-band, assuming an observation angle of 34°, have been initially considered to evaluate the reliability of the methodology. The approach has been also extended by considering the incidence angle as an additional input parameter to make the derived model applicable for terrains with complex topography. The use of neural networks has considerably decreased the computational time required by the IEMM.

It has been proved that the neural networks we have designed reproduce the behavior of IEMM fairly well both for flat and for tilted surfaces. The correlation between IEMM- and NN-derived backscattering coefficients has turned out to be close to the unit and we have also found an almost null mean error and a root mean square error not exceeding 1.3 dB at L-band and 0.7 dB at C-band (considering a variable incidence angle). A simple theoretical exercise has also indicated that the use of the trained networks within an iterative retrieval algorithm can be suitable for a typical problem such as retrieving soil moisture from radar data.

It is worth noting that the proposed approach can also be used to train the network on a database merging both model outputs and real measurements, thus providing a way to deal with the uncertainties of any theoretical model.

## Figures and Tables

**Table 1.** Number of epochs (*N_E_*) ensuring the convergence of the training algorithm (*N_r_* = 500) and values of the objective function (*E_R_*) at the final epoch for different numbers of hidden neurons (*N_h_*). The NN designed for L-band, *θ_i_* = 34° (*i.e., N_i_* = 3), with one hidden layer is considered.

***N****_h_*	***N****_E_*	***E****_R_*
5	79	10.5
10	134	5.4
15	286	3.1
20	343	2.9
25	545	2.5

**Table 2.** Same as Table 1, but for two hidden layers of *N_h_*_1_ and *N_h_*_2_ neurons, respectively.

***N****_h_***_1_**	***N****_h_***_2_**	***N****_E_*	***E****_R_*
8	7	380	0.56
12	7	420	0.29
12	10	440	0.17
15	7	645	0.10
15	10	920	0.04
17	12	1,350	0.03

**Table 3.** Same as Table 2, but for *N_i_* = 4 (*i.e., θ_i_* as an additional input parameter). *N_r_* = 2,000.

***N****_h_***_1_**	***N****_h_***_2_**	***N****_E_*	***E****_R_*
15	10	780	2.81
20	15	962	0.77
25	20	1,202	0.32
30	25	1,580	0.06

**Table 4.** Comparison between IEMM- and NN-derived *σ*^0^ in terms of correlation coefficient *ρ*, bias error *b* and root mean square error *rmse*.

	**Vertical polarization**		**Horizontal polarization**	

	L-band	C-band	L-band	C-band
***ρ***	0.99	1.00	0.99	1.00
***b* [dB]**	−0.08	−0.01	−0.02	−0.01
***rmse* [dB]**	0.66	0.31	0.78	0.27

**Table 5.** Same as Table 4, but for *θ_i_* assumed as an additional input parameter.

	**Vertical polarization**		**Horizontal polarization**	

	L-band	C-band	L-band	C-band
***ρ***	0.95	0.99	0.98	1.00
***b* [dB]**	0.05	−0.03	0.05	0.01
***rmse* [dB]**	1.21	0.66	1.26	0.52

**Table 6.** Results of the comparison between the soil moistures belonging to the test sets built for fixed *θ_i_* (500 records) and those estimated by using NNs in the retrieval algorithm based on (13).

	**L-band**	**C-band**
***ρ***	0.88	0.74
***b* [m^3^/m^3^]**	0.01	0.01
***rmse* [m^3^/m^3^]**	0.05	0.07
